# Radiomics and deep learning for intracranial aneurysm rupture-status assessment on CTA/MRA: a multicenter retrospective study

**DOI:** 10.3389/fnins.2026.1856293

**Published:** 2026-09-03

**Authors:** Tao Wang, Jiang Fan, Yi He, Changren Huang, Kunyang Bao

**Affiliations:** 1 Department of Neurosurgery, The Affiliated Hospital of Southwest Medical University, Luzhou, Sichuan, China; 2 Neurosurgical Clinical Research Center of Sichuan Province, Luzhou, China; 3 Laboratory of Neurological Diseases and Brain Functions, The Affiliated Hospital of Southwest Medical University, Luzhou, China

**Keywords:** CTA, deep learning, intracranial aneurysm, machine learning, MRA, neuroimaging methods, radiomics, rupture status

## Abstract

**Background:**

Rupture of intracranial aneurysms leads to aneurysmal subarachnoid hemorrhage with severe adverse outcomes. Current retrospective imaging studies merely differentiate existing ruptured and unruptured aneurysms instead of forecasting prospective rupture risk. To address this limitation, we constructed a multimodal fusion model combining handcrafted radiomics and deep learning embeddings from routine CTA/MRA for aneurysm rupture discrimination and performed internal validation.

**Methods:**

This multicenter retrospective study screened 520 patients with 572 saccular aneurysms. After eligibility and imaging quality control, 400 patients with 400 index aneurysms were enrolled. Patients were divided into training, validation and test sets (70/15/15) with balanced ruptured/unruptured aneurysm ratios. A total of 1,316 radiomic features were extracted from aneurysm segmentations; stable, non-redundant predictors (n=42) were retained via filtering and LASSO regression. A 3D ResNet produced 512-dimensional deep embeddings, compressed to 128 components by PCA. We fused radiomic and deep features as a 170-dimensional input for an XGBoost classifier. Model performance was assessed via discrimination, calibration and decision-curve analysis.

**Results:**

The fusion model reached an AUC of 0.928, accuracy 0.889, sensitivity 0.902, specificity 0.872, PPV 0.886 and NPV 0.889 on the test set, superior to single radiomic and deep learning models. The model exhibited excellent calibration (slope=0.98, Brier score=0.082). Decision-curve analysis demonstrated greater net clinical benefit across probability thresholds of 0.10–0.70.

**Discussion:**

Integrating reproducible radiomic metrics and volumetric deep embeddings significantly improves the discrimination of aneurysm rupture status. Nevertheless, all rupture labels were determined at initial presentation, and external independent validation was absent. Thus, this pipeline is only an internally validated methodological tool for imaging phenotype analysis, rather than a ready-to-use clinical model for predicting future aneurysm rupture.

## 1 Introduction

### 1.1 Background and clinical significance

Intracranial aneurysms affect approximately 3–5% of adults, whereas annual rupture risk is relatively low and varies according to patient-level and aneurysm-specific characteristics (Korja et al., 2014; Kleinloog et al., 2018). When rupture occurs, aneurysmal subarachnoid hemorrhage is associated with substantial mortality and long-term neurological disability, making individualized assessment central to surveillance and treatment planning (Thompson et al., 2015). Current clinical practice therefore requires models that can refine risk evaluation beyond aneurysm size alone while remaining reproducible across imaging settings.

Conventional clinical scores provide useful but incomplete guidance. The PHASES score estimates 5-year rupture risk using population, hypertension, age, aneurysm size, earlier subarachnoid hemorrhage, and aneurysm site, but it does not explicitly incorporate detailed aneurysm morphology, wall-related imaging phenotypes, or hemodynamic context (Greving et al., 2014; Thompson et al., 2015). External validation in patients with multiple intracranial aneurysms showed limited discrimination, with patient-level AUC 0.57 (Feng et al., 2021). The Unruptured Intracranial Aneurysm Treatment Score (UIATS) integrates more than 30 clinical and imaging factors to structure treatment recommendations, but its consensus-based nature introduces subjectivity and variable agreement between observers (Etminan et al., 2015). External studies have reported modest performance in different tasks: Ravindra et al. found AUC 0.646 for agreement with real-world treatment decisions, whereas Molenberg et al. reported AUC 0.62 for predicting aneurysm growth or rupture during follow-up (Ravindra et al., 2018; Molenberg et al., 2021).

Quantitative imaging offers an opportunity to capture aneurysm phenotypes more comprehensively. Radiomics converts medical images into high-dimensional descriptors of shape, intensity distribution, and texture heterogeneity, thereby encoding subtle morphological and structural signals that may not be fully captured by conventional measurements (Aerts et al., 2014; Kumar et al., 2012; Gillies et al., 2016; Lambin et al., 2017). Deep learning can further learn hierarchical spatial representations directly from angiographic volumes and has shown promise in aneurysm detection, segmentation, and rupture-status assessment on CTA and MRA (Sichtermann et al., 2019; Li et al., 2023; Xie et al., 2023; Choi et al., 2025). Recent systematic reviews and meta-analyses indicate that radiomics- and AI-based models often achieve encouraging discrimination, but they also emphasize substantial heterogeneity, inconsistent external validation, and risk of optimistic performance estimates in retrospective studies (Sohrabi-Ashlaghi et al., 2024; Wang et al., 2024; Zhong et al., 2024; Daga et al., 2025; Zhang et al., 2026).

A key methodological concern is that many existing studies, including the present work, compare aneurysms that are already ruptured at presentation with unruptured aneurysms. Such designs evaluate rupture-status discrimination rather than prospective rupture prediction. Because aneurysm geometry and image appearance may change after rupture, retrospective labels can introduce reverse-causality bias and confounding by established risk factors such as size, location, hypertension, and age. Therefore, imaging-based classifiers should be interpreted as tools for characterizing rupture-associated phenotypes unless validated prospectively in unruptured aneurysm cohorts with longitudinal outcomes.

Against this background, this study developed an integrated radiomics–deep learning framework for multicenter CTA/MRA-based rupture-status assessment of saccular intracranial aneurysms. The study evaluates whether combining reproducible handcrafted features with learned volumetric embeddings improves internal discrimination, calibration, and decision-curve performance compared with single-modality imaging models, while explicitly acknowledging that external and prospective validation is required before clinical risk-prediction claims can be made.

### 1.2 Study objectives

This study aimed to develop and internally evaluate a multicenter imaging-based model for rupture-status discrimination in saccular intracranial aneurysms using radiomic features and deep-learning embeddings extracted from routine CTA/MRA. The primary hypothesis was that early fusion of reproducible radiomic descriptors and learned volumetric representations would improve internal discrimination and calibration compared with single-modality imaging models. Because rupture status was defined at presentation, the study was not designed to prove prospective prediction of future rupture.

The specific objectives were as follows:

1. To assemble a multicenter retrospective CTA/MRA cohort of saccular intracranial aneurysms with adjudicated rupture status at presentation and standardized preprocessing across participating centers.
2. To extract a high-dimensional radiomic panel (*n* = 1,316) from aneurysm-sac segmentations and apply stability-oriented feature selection, including ICC filtering, redundancy pruning, and LASSO, to identify reproducible imaging predictors.
3. To derive high-level volumetric representations using a three-dimensional residual convolutional neural network trained under patient-level data separation, with validation and test data kept separate from model fitting.
4. To construct an early-fusion classifier by combining selected radiomic features and PCA-compressed deep embeddings, and to compare its internal test performance with radiomics-only and deep-learning–only models using AUC, accuracy, sensitivity, specificity, PPV, NPV, calibration, and decision-curve analysis.
5. To interpret model findings cautiously in the context of retrospective rupture-status classification and identify methodological requirements for future work, including independent external validation, prospective outcome assessment, segmentation-uncertainty analysis, and comparison with established clinical scores where complete PHASES/UIATS variables are available.

This work is intended as a technical and methodological step toward imaging-based neurovascular risk stratification, rather than a deployable clinical decision tool. Prospective validation in independent cohorts of unruptured aneurysms is necessary before the model can be used to guide treatment escalation or surveillance intervals.

## 2 Materials and methods

### 2.1 Study cohort and imaging data

This multicenter retrospective study was assembled from harmonized clinical and imaging records collected at three tertiary neurovascular centers between January 2018 and December 2024. The initial screening set included 520 patients with 572 intracranial aneurysms identified from institutional neurovascular imaging databases and confirmed by digital subtraction angiography (DSA), computed tomography angiography (CTA), or magnetic resonance angiography (MRA). Rupture status was assigned according to presentation imaging, operative records, and clinical adjudication. Therefore, the present analysis should be interpreted as rupture-status discrimination rather than prospective prediction of future rupture. This distinction is important because aneurysm morphology and imaging appearance may change after rupture, introducing potential reverse-causality bias in retrospective ruptured-versus-unruptured comparisons (Korja et al., 2014; Kleinloog et al., 2018; Thompson et al., 2015).

Eligibility criteria were as follows: (1) age ≥18 years; (2) diagnosis of an intracranial aneurysm on CTA, MRA, or DSA; (3) saccular aneurysm morphology after eligibility review; (4) available high-resolution angiographic imaging suitable for aneurysm segmentation and quantitative feature extraction; and (5) adjudicated rupture status at presentation. Cases were excluded for fusiform, dissecting, or mycotic aneurysm morphology (*n* = 18), prior subarachnoid hemorrhage unrelated to the index lesion (*n* = 12), poor imaging quality caused by motion, metal artifact, or inadequate vascular opacification (*n* = 28), or incomplete imaging metadata required for standardized preprocessing and imaging-based model development (*n* = 10). After eligibility screening, 452 patients with 500 saccular intracranial aneurysms and adjudicated rupture status were retained as the eligible analytic cohort.

A second imaging and preprocessing quality-control step was applied before model development. Fifty-two patients were excluded because of incomplete paired radiomics/deep-learning feature availability (*n* = 17), failed or unstable aneurysm segmentation quality control (*n* = 12), inadequate aneurysm-centered patch coverage for deep-learning input (*n* = 10), residual preprocessing or registration failure (*n* = 7), or missing variables required for split-level stratification or model evaluation (*n* = 6). The final modeling cohort therefore included 400 patients. To maintain patient-level independence, one index aneurysm was selected per patient for model development. For patients presenting with aneurysmal rupture and multiple aneurysms, the clinically adjudicated ruptured aneurysm was selected as the index lesion. For patients with multiple unruptured aneurysms, the largest aneurysm was selected as the index lesion. Thus, the final modeling dataset consisted of 400 patients with 400 index aneurysms.

Data were split by patient into training, validation, and internal held-out test sets at a 70/15/15 ratio. The training set included 280 patients, the validation set included 60 patients, and the internal held-out test set included 60 patients. Rupture-status distributions were 112/168 ruptured/unruptured cases in the training set, 24/36 in the validation set, and 24/36 in the test set. All feature selection, dimensionality reduction, model fitting, hyperparameter tuning, and early stopping were performed without using the internal held-out test set.

All patients in the final modeling cohort had complete rupture-status labels, imaging modality metadata, aneurysm segmentation masks, radiomic feature availability, and deep-learning patch/embedding availability. Baseline demographic and clinical covariates were not used as inputs to the radiomics-only, deep-embedding, or integrated imaging models. Descriptive baseline analyses were therefore performed separately in the complete-covariate subset of the final modeling cohort. Among the 400 patients included in model development, 312 patients had complete demographic, clinical, and aneurysm-level variables for uniform baseline comparison, whereas 88 patients had at least one missing non-imaging baseline covariate. Specifically, hypertension status was missing in 54 patients, smoking history was missing in 64 patients, and 30 patients had both variables missing. These missing non-imaging covariates affected only the descriptive baseline table and did not affect imaging feature extraction, model training, validation, or internal held-out test evaluation. Variable-specific missing-data accounting is provided in Supplementary Supplementary Table S1.

**Figure 1:**
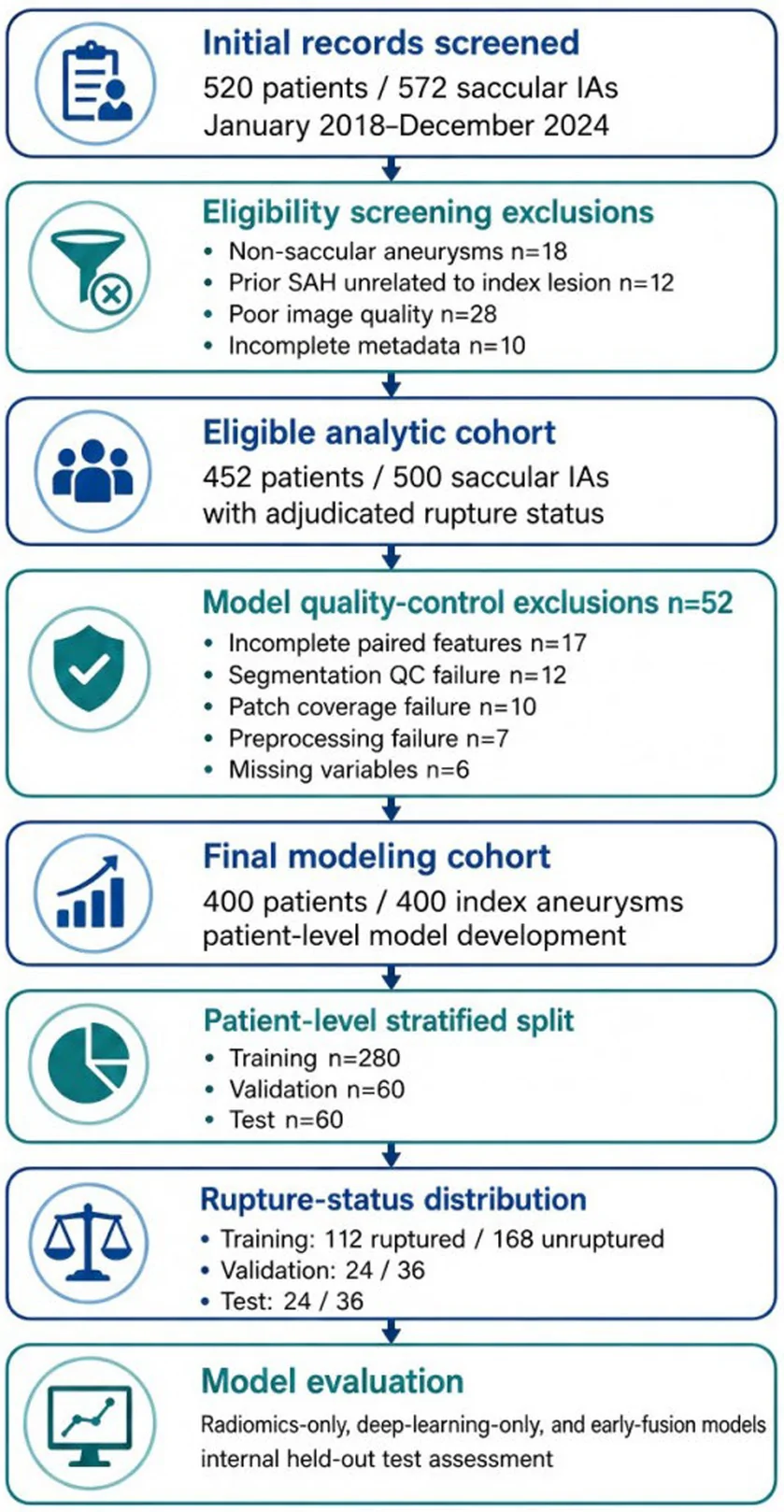
Patient selection and data processing pipeline.

All imaging studies were de-identified before analysis. The retrospective protocol was approved by the institutional review boards of the participating centers, and the requirement for written informed consent was waived because of the retrospective design and use of anonymized imaging and clinical data. No animal experiments were performed (Figure 1; Table 1).

**Table 1:** Summary of commonly used clinical scoring systems for intracranial aneurysm rupture-related assessment.

Scoring system	Predictors	Scoring range	Outcome measure	Reported AUC in external validation	References
PHASES	Population, hypertension, age, aneurysm size, earlier SAH, aneurysm site	0–22	Estimated 5-year rupture risk (%)	0.57^†^	Greving et al. (2014), Thompson et al. (2015), Feng et al. (2021)
UIATS	More than 30 factors including demographics, rupture-risk factors, treatment-risk factors, imaging features, and patient preference	N/A	Treatment recommendation; growth or rupture during follow-up in validation studies	0.646^‡^; 0.62^§^	Etminan et al. (2015), Ravindra et al. (2018), Molenberg et al. (2021)

^†^Patient-level discrimination in a multiple-aneurysm cohort. ^‡^Agreement with real-world treatment decisions in a single-center validation study. ^§^Prediction of aneurysm growth or rupture during follow-up in a prospective database study. SAH, subarachnoid hemorrhage; UIATS, Unruptured Intracranial Aneurysm Treatment Score.

### 2.2 Imaging protocol and preprocessing

CTA and MRA served as the primary imaging sources for radiomics extraction, deep-learning patch generation, and model development. DSA was used when available as a reference standard to support aneurysm diagnosis, anatomical localization, and rupture-status adjudication, but DSA images were not used as direct model inputs. This distinction was made to maintain consistency with the CTA/MRA-based scope of the study while allowing clinically adjudicated reference information to support cohort construction. Modality-specific acquisition settings are summarized in Table 2.

**Table 2:** Imaging acquisition protocols for CTA, MRA, and reference DSA in the multicenter study cohort.

Modality	Scanner model	Key acquisition parameters	Reconstruction parameters
CTA	Siemens SOMATOM Definition, GE Revolution CT (64–128 slice)	Tube voltage: 100–120 kVp; Tube current: 250–350 mA; Detector collimation: 0.6–0.625 mm; Matrix: 512 × 512; Contrast injection: 60–80 mL at 4–5 mL/s; Scan delay: bolus tracking	Slice thickness: 0.5–0.625 mm; Increment: 0.3 mm; Kernel: medium-sharp (B30f/Bp30)
MRA	Philips Ingenia, Siemens Magnetom Skyra (3.0 T)	Sequence: 3D TOF; TR: 20–25 ms; TE: 3–4 ms; Flip angle: 18–20°; FOV: 200 × 200 mm; Matrix: 384 × 384; Isotropic voxel: 0.5–0.6 mm^3^	Reconstruction: MIP & source data, slice thickness 0.5 mm, spacing 0.5 mm
DSA	Philips Allura Xper FD20 (biplane)	Frame rate: 3–6 fps; Projection: orthogonal & rotational; Contrast injection: 5–7 mL/s, total 10–14 mL; Catheter size: 5–6F; FOV: 17–22 cm	Reconstruction: subtracted frames, pixel size 0.15–0.2 mm

CTA examinations were performed on 64–128-slice multidetector CT systems, including Siemens SOMATOM Definition and GE Revolution CT scanners. Tube voltage ranged from 100 to 120 kVp, tube current ranged from 250 to 350 mA, detector collimation was 0.6–0.625 mm, and the acquisition matrix was 512 × 512. Contrast medium was injected at 4–5 mL/s with a total volume of 60–80 mL, and bolus tracking was used to optimize arterial-phase timing. Images were reconstructed with a medium-sharp kernel at a slice thickness of 0.5–0.625 mm and an increment of approximately 0.3 mm. These reconstruction settings were recorded because CT acquisition and reconstruction parameters can influence radiomic feature repeatability and cross-scanner comparability (Mackin et al., 2015; Choe et al., 2019).

MRA examinations were acquired on 3.0-T scanners, including Philips Ingenia and Siemens Magnetom Skyra systems, using three-dimensional time-of-flight sequences. Typical acquisition parameters included TR of 20–25 ms, TE of 3–4 ms, flip angle of 18–20°, field of view of approximately 200 × 200 mm, acquisition matrix of 384 × 384, and isotropic voxel size of 0.5–0.6 mm^3^. Source images were used for segmentation and feature extraction; maximum-intensity projection images were reviewed for anatomical confirmation but were not used as primary quantitative inputs. High-resolution TOF-MRA has been widely used for intracranial aneurysm detection, segmentation, and automated vascular image analysis (Sichtermann et al., 2019; Yamanouchi et al., 2022).

DSA was performed using biplane angiographic systems, including Philips Allura Xper FD20, with orthogonal and rotational projections acquired at 3–6 frames/s. Contrast was injected at 5–7 mL/s with a total volume of 10–14 mL, catheter size was typically 5–6F, and reconstructed pixel size ranged from 0.15 to 0.2 mm. When available, DSA was used to support confirmation of aneurysm morphology, location, and rupture-related clinical adjudication. It was not used to extract radiomic or deep-learning features for the primary CTA/MRA model.

All CTA and MRA volumes underwent standardized preprocessing before segmentation and feature extraction. CTA and MRA intensities were normalized to a common 0–255 range after modality-specific preprocessing. Volumes were resampled to 0.5-mm isotropic voxel spacing and aligned to a common anatomical orientation. When more than one imaging modality was available for the same patient, the CTA or MRA study with the highest segmentation quality and closest temporal relationship to presentation was selected as the quantitative model input. Rigid registration was performed only when required for anatomical review or segmentation consensus. Fixed voxel geometry, transparent preprocessing, and consistent image handling were applied to reduce variability in downstream radiomic and deep-learning features (Traverso et al., 2018; Zwanenburg et al., 2020).

Aneurysm sacs were manually segmented on CTA or MRA source images using ITK-SNAP by two board-certified neuroradiologists with 8 and 12 years of neurovascular imaging experience, respectively. ITK-SNAP has been widely used for interactive three-dimensional medical image segmentation (Yushkevich et al., 2006). Segmentations were performed on the aneurysm sac, and the parent artery was excluded whenever anatomically separable. Source images were reviewed together with multiplanar reconstructions, and DSA information was consulted when available to support anatomical localization and rupture-status adjudication. Disagreements between readers were resolved by consensus review. The consensus aneurysm-sac mask was used for radiomic feature extraction, aneurysm-centered patch generation, and final model development.

To assess segmentation-related uncertainty, 20% of the final modeling cohort was randomly selected for repeated independent segmentation, corresponding to 80 of the 400 patients. Repeated segmentation cases were selected to preserve representation of rupture status and imaging modality where feasible. Inter-reader mask agreement was quantified using the Dice similarity coefficient. Feature-level reproducibility was assessed using the intraclass correlation coefficient (ICC) calculated between radiomic features extracted from the original and repeated segmentations. For leakage-aware feature selection, reproducibility filtering was fitted within the training partition only, and radiomic descriptors with ICC > 0.85 were retained. The resulting reproducibility filter was then applied unchanged to validation and internal held-out test data. Summary Dice and ICC values from the repeated-segmentation analysis are reported in the Results section.

### 2.3 Radiomic feature extraction

Radiomic features were extracted from preprocessed CTA or MRA source volumes using the consensus aneurysm-sac masks described above. DSA was not used for radiomic feature extraction. Feature extraction was performed using PyRadiomics version 3.0.1 implemented in Python 3.10. Feature definitions followed the Image Biomarker Standardization Initiative principles where applicable (van Griethuysen et al., 2017; Zwanenburg et al., 2020). Before feature extraction, all quantitative input images had been resampled to 0.5-mm isotropic voxel spacing and normalized as described in Section 2.2. Gray-level discretization was performed using a fixed bin width of 25 for CTA and MRA after intensity normalization. The same extraction settings were applied across centers to reduce protocol-related variation.

A total of 1,316 radiomic descriptors were computed for each index aneurysm. These included 14 shape features, 18 first-order intensity statistics, 75 original texture features, and 1,209 wavelet- or Laplacian-of-Gaussian (LoG)-derived features. The original texture features comprised gray-level co-occurrence matrix, gray-level run-length matrix, gray-level size-zone matrix, gray-level dependence matrix, and neighboring gray-tone difference matrix descriptors. Radiomic feature families were intended to capture complementary aspects of aneurysm geometry, intensity distribution, and texture heterogeneity (Aerts et al., 2014; Kumar et al., 2012; Gillies et al., 2016; Lambin et al., 2017; Tomaszewski and Gillies, 2021). Wavelet features were generated using eight three-dimensional high-pass/low-pass decomposition combinations, including LLL, LLH, LHL, LHH, HLL, HLH, HHL, and HHH. LoG-filtered features were generated using sigma values of 1.0, 2.0, 3.0, 4.0, and 5.0 mm to capture fine-to-coarse local heterogeneity patterns. The extracted feature categories are summarized in Table 3.

**Table 3:** Categories and definitions of extracted radiomic features.

Feature family	Number of features	Examples	Definition/extraction setting
Shape	14	Volume, maximum 3D diameter, surface area, compactness, sphericity	Describes aneurysm geometry and surface configuration from the segmented aneurysm sac.
First-order statistics	18	Mean, variance, skewness, kurtosis, entropy	Quantifies voxel-intensity distribution within the aneurysm sac without considering spatial relationships.
GLCM	24	Contrast, correlation, energy, homogeneity	Describes gray-level co-occurrence and local intensity-pair relationships.
GLRLM	16	Short-run emphasis, long-run emphasis, run-length non-uniformity	Captures consecutive voxel runs with similar gray-level values.
GLSZM	16	Small-zone emphasis, large-zone emphasis, zone entropy	Measures connected zones with the same gray-level intensity.
GLDM	14	Dependence non-uniformity, dependence entropy	Characterizes gray-level dependence within local neighborhoods.
NGTDM	5	Coarseness, contrast, busyness, complexity, strength	Describes local intensity differences between voxels and neighboring regions.
Wavelet- and LoG-derived features	1,209	Wavelet LLL/LLH/LHL/LHH/HLL/HLH/HHL/HHH; LoG σ = 1.0–5.0 mm	Multi-scale transformed features derived from first-order and texture descriptors to capture fine-to-coarse heterogeneity patterns.

GLCM, gray-level co-occurrence matrix; GLRLM, gray-level run-length matrix; GLSZM, gray-level size-zone matrix; GLDM, gray-level dependence matrix; NGTDM, neighboring gray-tone difference matrix; LoG, Laplacian-of-Gaussian. Radiomic features were extracted from CTA or MRA source images using consensus aneurysm-sac masks; DSA was not used for radiomic feature extraction.

To minimize information leakage, all radiomic preprocessing parameters used for model development, including feature scaling, reproducibility filtering, redundancy pruning, and supervised feature selection, were fitted within the training partition only. Feature reproducibility was evaluated using repeated segmentation data. For leakage-aware feature filtering, the intraclass correlation coefficient (ICC) threshold was estimated using only repeated-segmentation cases within the training partition. Radiomic descriptors with ICC > 0.85 were retained. Repeated-segmentation results across the full repeated-segmentation subset are reported descriptively in the Results section to characterize segmentation uncertainty.

After reproducibility filtering, highly collinear descriptors were removed within the training set using a Pearson correlation threshold of |r| ≥ 0.90. When two features exceeded this threshold, the feature with lower univariate stability or higher redundancy with other retained descriptors was removed. The resulting training-set feature list and preprocessing transformations were then applied unchanged to the validation and internal held-out test sets. This stability and redundancy filtering procedure reduced the initial 1,316 descriptors to 187 candidate radiomic features.

**Figure 2:**
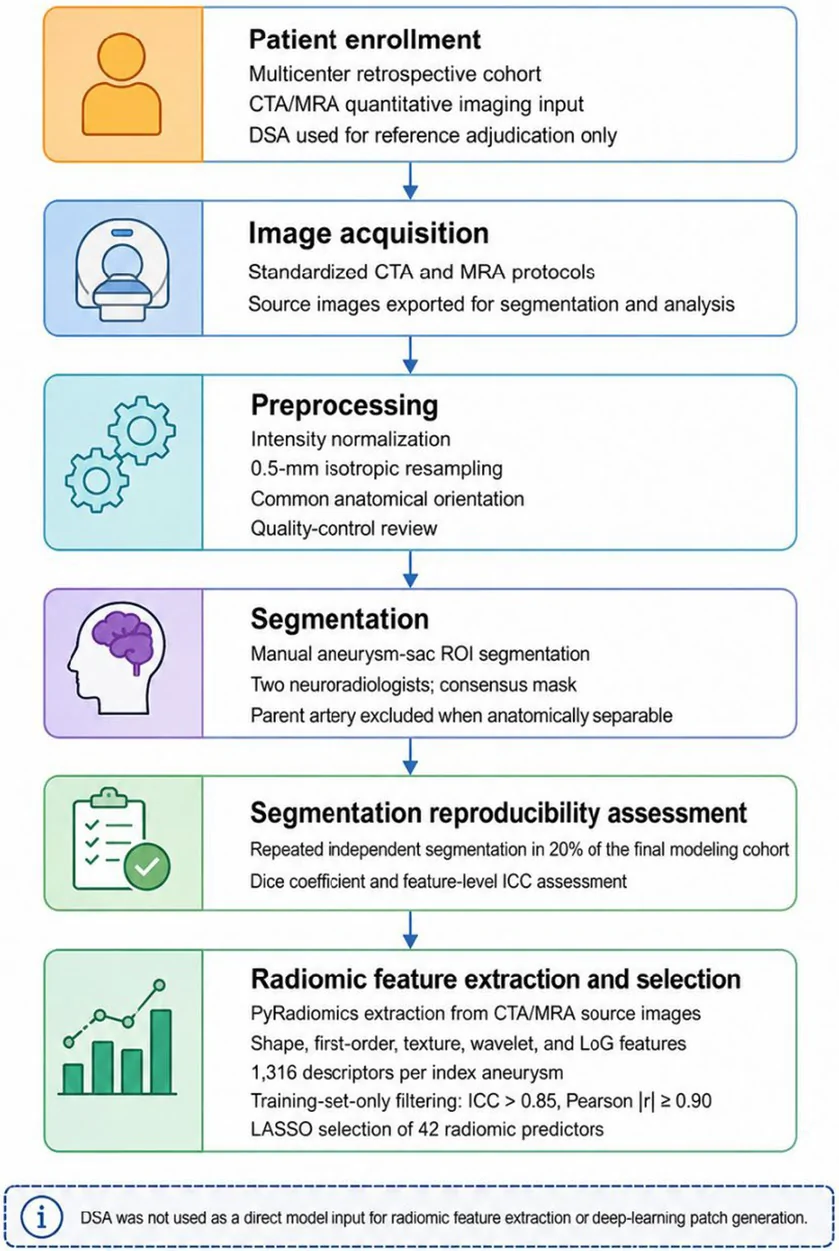
Imaging preprocessing workflow.

Final supervised radiomic feature selection was performed using LASSO logistic regression within nested cross-validation on the training set only. The regularization parameter was selected using the one-standard-error rule to favor a sparse and more stable feature set. This procedure yielded 42 radiomic predictors for downstream radiomics-only and early-fusion modeling. No validation-set or internal held-out test-set outcome labels were used during radiomic feature filtering, LASSO selection, or transformation fitting. Such stability-oriented and leakage-aware feature selection is essential for improving the credibility and reproducibility of radiomics-based modeling studies (Traverso et al., 2018; Lambin et al., 2017) (Figure 2).

### 2.4 Deep learning feature extraction

To capture high-level spatial representations beyond handcrafted radiomic descriptors, a custom three-dimensional residual convolutional neural network was implemented for aneurysm-centered patch analysis. CTA or MRA source volumes served as the quantitative inputs for deep-learning patch generation. DSA was not used as a direct deep-learning input. The term “custom 3D residual CNN” is used because the implemented architecture comprised three Conv3D blocks and two residual blocks rather than the full standard ResNet-18 configuration. Three-dimensional CNNs have increasingly been used for intracranial aneurysm detection, segmentation, and rupture-status assessment because they can learn hierarchical spatial features directly from volumetric angiographic data (Sichtermann et al., 2019; Li et al., 2023; Xie et al., 2023; Choi et al., 2025).

Aneurysm-centered patches were generated from preprocessed CTA or MRA volumes using the centroid of the consensus aneurysm-sac mask as the patch center. The aneurysm bounding box was expanded by an 8-mm margin to include local neck–dome configuration and adjacent vascular context. Each patch was resampled, cropped, or zero-padded to 64 × 64 × 64 voxels at 0.5-mm isotropic spacing, corresponding to a 32-mm cubic field of view. Patch extraction was performed after patient-level data splitting, and the same deterministic patch-generation algorithm was applied to the training, validation, and internal held-out test sets. No patch from a patient in the validation or test set was used during CNN training.

The network consisted of three Conv3D + BatchNorm + ReLU blocks with 64, 128, and 256 filters, respectively, followed by two residual blocks, global average pooling, and a 512-unit fully connected layer with dropout. A binary rupture-status classification head was attached during CNN training. The model was initialized with Xavier uniform weights and optimized using Adam with an initial learning rate of 1 × 10^−4^, batch size of 16, and class-weighted cross-entropy loss. Class weights were calculated from the training set only. Data augmentation was applied only to the training set and included random rotations within ±10°, axial and sagittal flips, random intensity scaling, and low-amplitude Gaussian noise. These augmentation strategies were used to reduce overfitting and improve robustness to scanner- and protocol-related variation (Choe et al., 2019).

Training was performed for a maximum of 150 epochs, with early stopping based on validation loss. The validation set was used only for early stopping and hyperparameter selection, whereas the internal held-out test set was not used during CNN training, augmentation-parameter selection, early stopping, feature extraction tuning, PCA fitting, or downstream classifier development. After convergence, the standalone CNN classification head was evaluated separately on the internal held-out test set to determine whether the end-to-end CNN itself carried rupture-status information.

**Figure 3:**
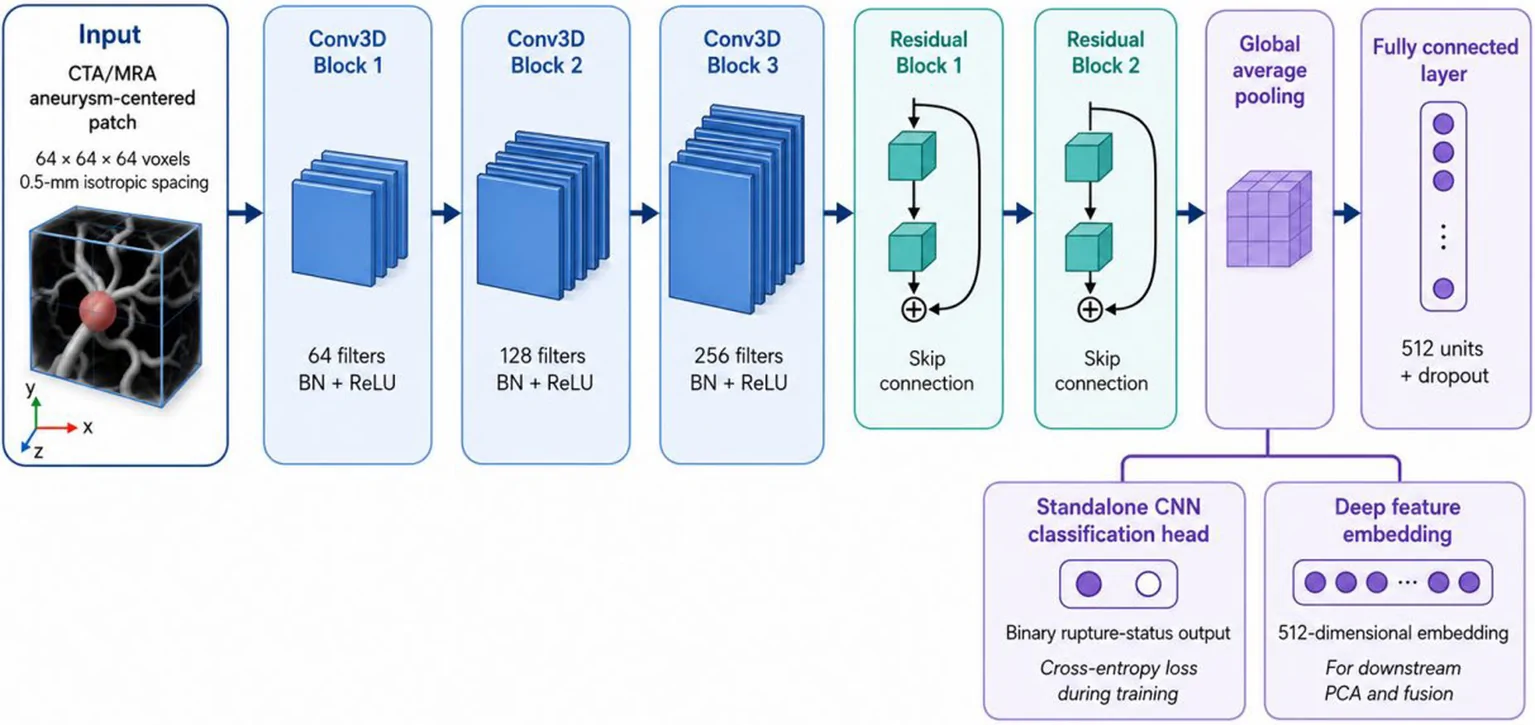
Custom 3D residual CNN for deep feature extraction.

For embedding-based modeling, the trained CNN was frozen after convergence, the classification head was removed, and 512-dimensional embeddings were extracted from the penultimate fully connected layer. The frozen CNN was then used without further updating to generate embeddings for the validation and internal held-out test sets. Principal component analysis was fitted only on the training-set embeddings and then applied unchanged to validation and test embeddings. Components explaining at least 95% of the training-set variance were retained, resulting in a 128-dimensional compact deep-feature vector for each index aneurysm. These PCA-compressed deep embeddings were used for the deep-embedding model and for subsequent early fusion with the selected radiomic predictors (Figure 3).

### 2.5 Integration of radiomics and deep learning features

The final radiomic feature vector contained 42 LASSO-selected predictors derived from the training-set-only feature-selection pipeline. The deep-learning feature vector contained 128 PCA-compressed components derived from the frozen custom 3D residual CNN. These two feature groups were concatenated for each patient to form a 170-dimensional early-fusion representation, consisting of 42 handcrafted radiomic features and 128 learned deep components. Fusion strategies combining engineered radiomic descriptors and learned deep representations have been proposed to improve aneurysm rupture-status assessment compared with single-domain models, although external validation remains limited across the field (Ye et al., 2024; Turhon et al., 2023; Wang et al., 2024; Jia et al., 2025).

A gradient-boosting classifier implemented with XGBoost was trained on the fused feature space (Chen and Guestrin, 2016). XGBoost was selected because it can model nonlinear feature interactions, handle mixed feature distributions, and incorporate regularization to reduce overfitting in structured biomedical prediction tasks. All transformation and model-development steps, including feature scaling, radiomic feature selection, PCA fitting, feature concatenation, and classifier hyperparameter tuning, were performed using the training partition only. The validation and internal held-out test sets were transformed using the fitted training-set parameters without refitting or outcome-label access.

Hyperparameters for the XGBoost classifier were selected by five-fold cross-validation within the training set. The search space included maximum depth {2, 3, 4, 5}, learning rate {0.01, 0.03, 0.05, 0.10}, number of estimators {100, 200, 300, 500}, subsample ratio {0.7, 0.8, 1.0}, column-sampling ratio {0.7, 0.8, 1.0}, minimum child weight {1, 3, 5}, L1 regularization {0, 0.01, 0.1}, and L2 regularization {1, 5, 10}. The final selected configuration was maximum depth 3, learning rate 0.03, 300 estimators, subsample ratio 0.8, column-sampling ratio 0.8, minimum child weight 3, L1 regularization 0.01, and L2 regularization 5. A fixed random seed of 2026 was used for patient-level data splitting, CNN initialization, cross-validation, PCA fitting, and XGBoost training to improve reproducibility.

The integrated model generated rupture-status probability estimates for the internal held-out test set. These estimates were used for discrimination analysis, calibration assessment, and decision-curve analysis. Because the outcome label reflected rupture status at clinical presentation, the model output should be interpreted as an internally evaluated rupture-status probability estimate rather than a prospectively validated rupture-risk probability.

**Figure 4:**
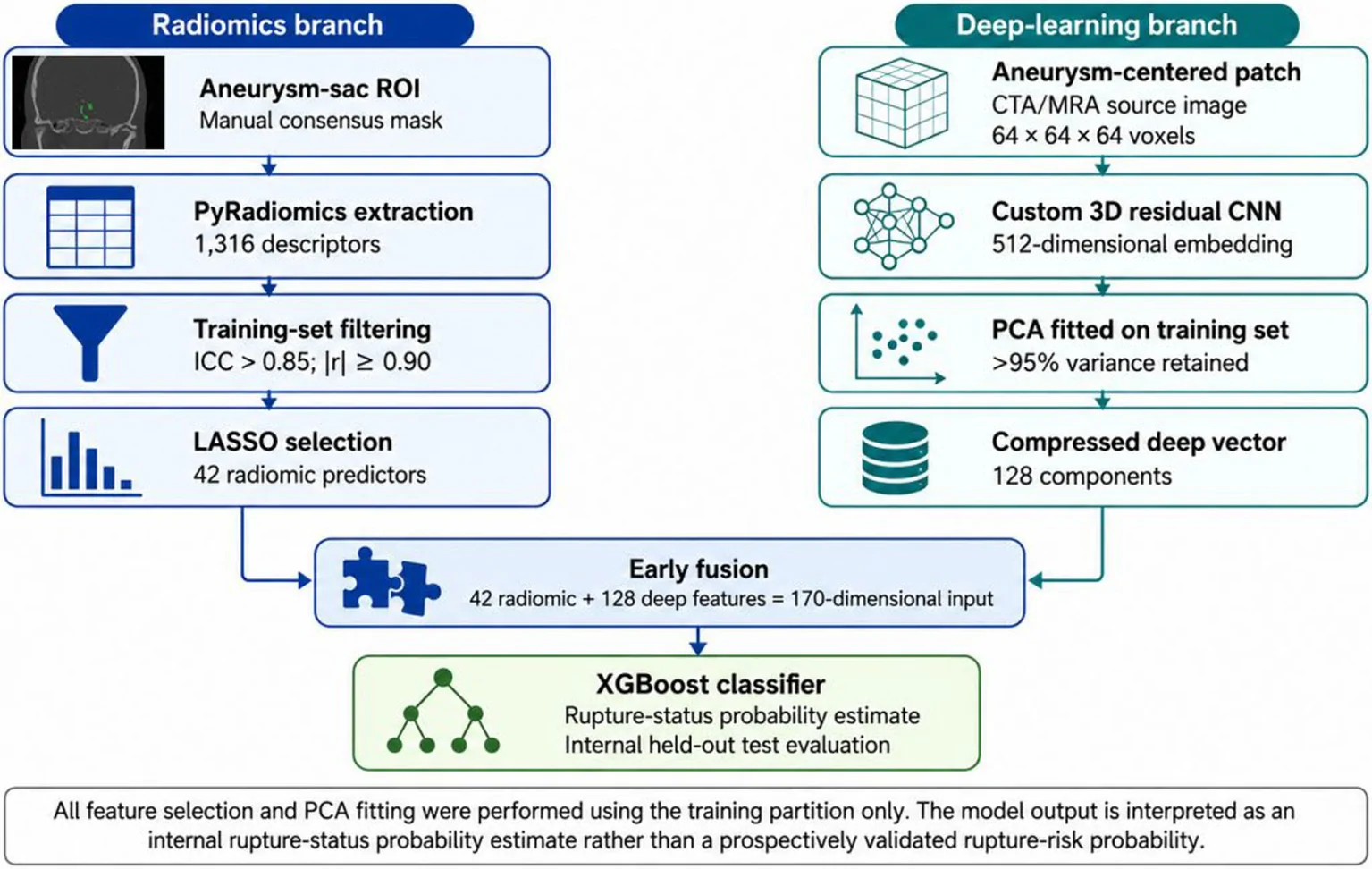
Early-fusion workflow combining radiomics and deep learning features.

Feature attribution was performed using model-based importance ranking and SHAP analysis to evaluate the relative contributions of radiomic variables and PCA-compressed deep components. SHAP provides an additive feature-attribution framework for interpreting model predictions (Lundberg and Lee, 2017). Because PCA-compressed deep components are not directly interpretable anatomical variables, interpretability was considered partial and was anchored primarily in selected radiomic features, domain-level feature contributions, and post-hoc attribution analysis (Figure 4).

### 2.6 Model training and internal validation

The final modeling cohort included 400 patients, each represented by one index aneurysm. Patient-level splitting was used to prevent data leakage from multiple aneurysms belonging to the same individual. The training set included 280 patients, the validation set included 60 patients, and the internal held-out test set included 60 patients. Ruptured and unruptured cases were stratified across splits, resulting in 112/168 ruptured/unruptured cases in the training set, 24/36 in the validation set, and 24/36 in the internal held-out test set.

Four model configurations were evaluated. The standalone CNN model used the binary classification head attached to the custom 3D residual CNN. The radiomics-only model used the 42 LASSO-selected handcrafted radiomic predictors. The deep-embedding model used the 128 PCA-compressed embeddings extracted from the frozen CNN. The integrated model used the full 170-dimensional early-fusion representation, consisting of 42 radiomic predictors and 128 deep components. The standalone CNN classifier was evaluated separately to clarify the contribution of end-to-end deep classification, whereas the deep-embedding model assessed whether frozen CNN-derived representations benefited from downstream gradient-boosting classification.

Model performance was assessed on the internal held-out test set only after all feature selection, radiomic reproducibility filtering, redundancy pruning, LASSO selection, CNN training, early stopping, PCA fitting, hyperparameter tuning, and classifier fitting had been completed. The internal held-out test set was not used for model selection, transformation fitting, probability-threshold optimization, or recalibration. The primary discrimination metric was the area under the receiver-operating characteristic curve (AUC). Secondary metrics included accuracy, sensitivity, specificity, positive predictive value, negative predictive value, calibration slope, Brier score, and decision-curve net benefit.

**Figure 5:**
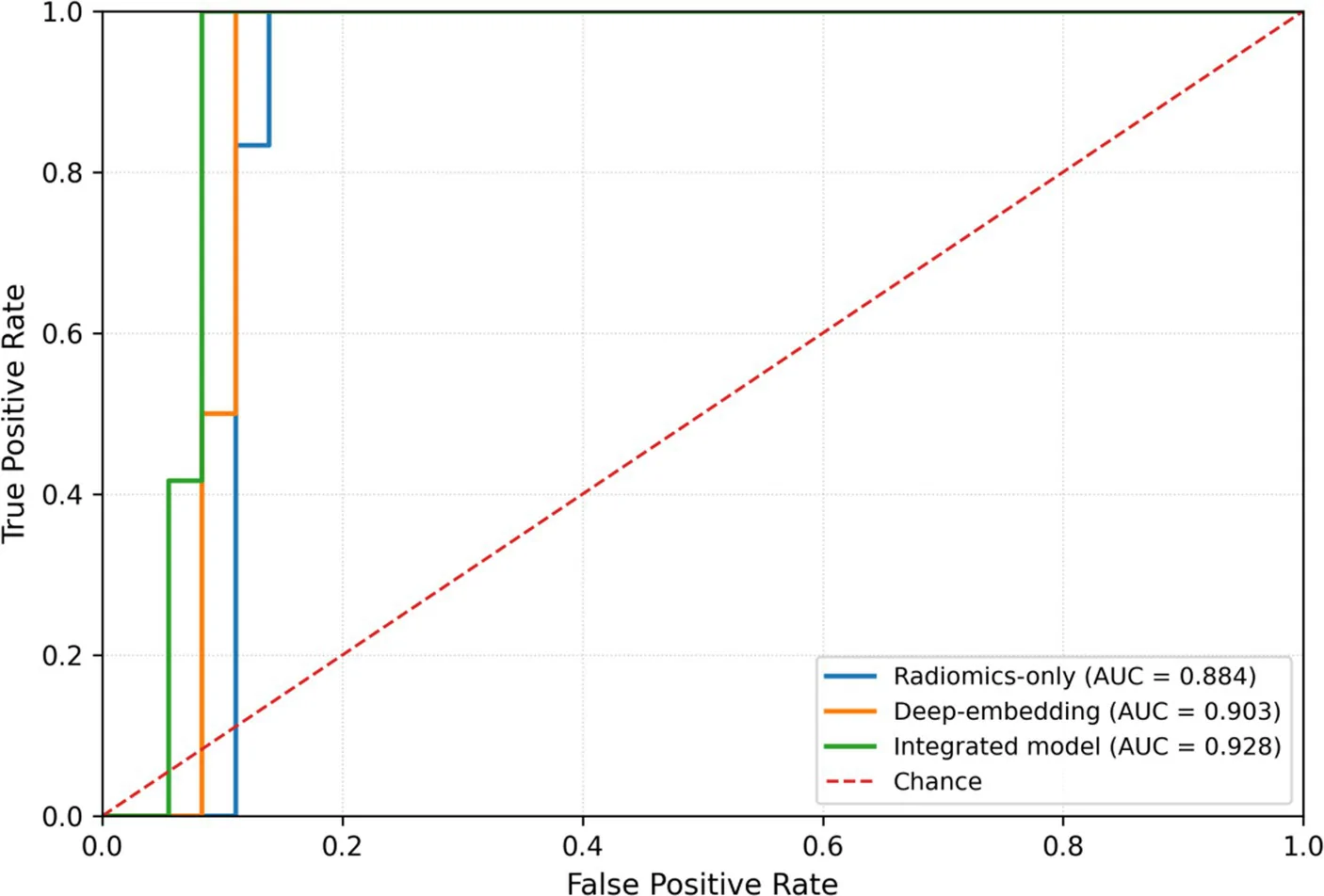
Empirical ROC curves comparing model performance on the internal held-out test set.

Receiver-operating characteristic curves were generated empirically from the binary rupture-status labels and model-predicted probability estimates in the internal held-out test set. No schematic ROC curves, manually smoothed curves, or curves redrawn to match target AUC values were used. AUC values reported in Table 4 were calculated from the same held-out test-set labels and predicted probabilities used to generate Figure 5. To support editorial and statistical verification, the de-identified internal held-out test-set labels and model-predicted probability estimates were retained as verification materials and can be provided to the editorial office or statistical reviewers upon reasonable request, subject to institutional data-sharing restrictions.

**Table 4:** Performance metrics of different models on the internal held-out test set.

Model	AUC (95% CI)	Accuracy (95% CI)	Sensitivity (95% CI)	Specificity (95% CI)	PPV (95% CI)	NPV (95% CI)
Standalone residual CNN	0.861 (0.779–0.931)	0.817 (0.717–0.900)	0.833 (0.667–0.958)	0.806 (0.667–0.917)	0.741 (0.571–0.889)	0.879 (0.750–0.971)
Radiomics-only model	0.884 (0.806–0.956)	0.861 (0.767–0.944)	0.854 (0.708–0.967)	0.868 (0.750–0.972)	0.873 (0.733–0.970)	0.849 (0.724–0.955)
Deep-embedding model	0.903 (0.829–0.966)	0.872 (0.783–0.950)	0.867 (0.727–0.971)	0.878 (0.764–0.972)	0.881 (0.747–0.974)	0.864 (0.743–0.957)
Integrated model	0.928 (0.862–0.978)	0.889 (0.800–0.967)	0.902 (0.771–1.000)	0.872 (0.750–0.972)	0.886 (0.755–0.978)	0.889 (0.769–0.976)

AUC values were calculated from empirical ROC curves generated using internal held-out test-set labels and model-predicted probabilities. Confidence intervals were estimated using 1,000 bootstrap resamples. Classification metrics are reported as bootstrap-resampled internal test estimates after applying validation-derived fixed operating thresholds. PPV, positive predictive value; NPV, negative predictive value; CNN, convolutional neural network.

Ninety-five percent confidence intervals for AUC and classification metrics were estimated using 1,000 bootstrap resamples of the internal held-out test set. Pairwise AUC comparisons between models were performed using the DeLong test (DeLong et al., 1988). Calibration was assessed using calibration plots, calibration slope, Brier score, and the Hosmer–Lemeshow goodness-of-fit test. Decision-curve analysis was performed across threshold probabilities from 0.10 to 0.70 and was interpreted as an internal exploratory analysis rather than as clinically validated decision thresholds. Because no independent external validation cohort was available, all model-performance estimates should be interpreted as internal validation results.

### 2.7 Statistical analysis

Statistical analyses were performed using R and Python. Descriptive baseline analyses were conducted in the complete-covariate subset of the final modeling cohort, as described in Section 2.1. Continuous variables were assessed for normality using the Shapiro–Wilk test. Normally distributed variables are reported as mean ± standard deviation, whereas non-normally distributed variables are reported as median and interquartile range. Categorical variables are summarized as counts and percentages. Between-group comparisons for ruptured and unruptured index aneurysms were performed using the Student t test or Mann–Whitney U test for continuous variables and the χ^2^ test or Fisher exact test for categorical variables, as appropriate. Because baseline clinical covariates were not used as model inputs, missing non-imaging covariates were not imputed for model development.

Receiver-operating characteristic analysis was performed using the pROC package in R and scikit-learn in Python. ROC curves were generated empirically from internal held-out test-set rupture-status labels and model-predicted probability estimates. No schematic, manually smoothed, or interpolated ROC curves were used. AUC values were calculated from the same held-out test-set labels and predicted probabilities used to generate the ROC curves. Ninety-five percent confidence intervals for AUC were estimated using 1,000 bootstrap resamples of the internal held-out test set. Pairwise AUC differences between models were evaluated using the DeLong test (DeLong et al., 1988).

Classification metrics included accuracy, sensitivity, specificity, positive predictive value, and negative predictive value. The operating threshold for each model was determined within the validation set and then fixed before application to the internal held-out test set. To account for the modest size of the internal held-out test set, point estimates and 95% confidence intervals for classification metrics were obtained using 1,000 bootstrap resamples of the test set after applying the fixed validation-derived threshold. Therefore, the reported classification metrics should be interpreted as bootstrap-resampled internal test estimates rather than as externally validated clinical operating characteristics.

Calibration was assessed using calibration plots, calibration slope, Brier score, and the Hosmer–Lemeshow goodness-of-fit test. Calibration plots were generated from internal held-out test-set predicted probabilities and observed rupture-status labels. The Brier score was used to quantify overall probabilistic accuracy, with lower values indicating better agreement between predicted probabilities and observed labels. Decision-curve analysis was performed to estimate net benefit across threshold probabilities from 0.10 to 0.70 (Vickers and Elkin, 2006). These thresholds were prespecified for internal exploratory evaluation and should not be interpreted as clinically validated treatment or surveillance thresholds.

All statistical tests were two-sided, and *p* < 0.05 was considered statistically significant. Given the retrospective design, modest internal test-set size, and absence of independent external validation, all model-performance statistics were interpreted as internal validation results.

## 3 Results

### 3.1 Patient characteristics

The final modeling cohort consisted of 400 patients with 400 index aneurysms. This cohort was used for radiomics extraction, deep-learning embedding generation, model training, validation, and internal held-out test evaluation. Baseline descriptive statistics were calculated in the complete-covariate subset of the final modeling cohort to ensure that all demographic, clinical, and aneurysm-level comparisons were performed on the same patients. This complete-covariate subset included 312 patients with 312 index aneurysms, comprising 128 ruptured aneurysms and 184 unruptured aneurysms. The remaining 88 patients had at least one missing non-imaging baseline covariate and were therefore excluded from the descriptive baseline table only. Specifically, hypertension status was missing in 54 patients, smoking history was missing in 64 patients, and 30 patients had both variables missing, as detailed in Supplementary Supplementary Table S1. These non-imaging covariates were not used as inputs to the radiomics-only, deep-embedding, or integrated imaging models and therefore did not affect model development or internal held-out test evaluation.

In the complete-covariate subset, the mean age was 56.8 ± 12.3 years, and 170 patients were women (54.5%). The ruptured group included 128 patients, and the unruptured group included 184 patients. Patients with ruptured aneurysms were modestly older than those with unruptured aneurysms (58.9 ± 11.4 vs. 55.3 ± 12.8 years, *p* = 0.018), and the proportion of female patients was higher in the ruptured group (61.7% vs. 49.5%, *p* = 0.041). Mean maximum aneurysm diameter was also greater in ruptured aneurysms than in unruptured aneurysms (8.6 ± 3.7 mm vs. 5.2 ± 2.4 mm, *p* < 0.001).

The most frequent aneurysm locations in the complete-covariate subset were the middle cerebral artery (MCA, 32.4%), internal carotid artery (ICA, 27.9%), anterior communicating artery (ACom, 21.5%), posterior communicating artery (PCom, 11.9%), and posterior circulation (6.4%). Location distributions differed between ruptured and unruptured aneurysms for MCA aneurysms (25.0% vs. 37.5%, *p* = 0.023), ICA aneurysms (21.9% vs. 32.1%, *p* = 0.049), and ACom aneurysms (31.3% vs. 14.7%, *p* = 0.002). PCom aneurysms and posterior circulation aneurysms did not differ significantly between groups (*p* = 0.312 and *p* = 0.394, respectively). Hypertension and smoking history showed borderline or non-significant differences between ruptured and unruptured groups (*p* = 0.058 and *p* = 0.088, respectively) and were not interpreted as statistically significant group differences in this cohort. These descriptive patterns are broadly consistent with established clinical and anatomical modifiers of aneurysm rupture status reported in population cohorts and practice guidelines (Korja et al., 2014; Kleinloog et al., 2018; Thompson et al., 2015).

### 3.2 Radiomic feature selection results

A total of 1,316 radiomic descriptors were extracted from each index aneurysm, including shape, first-order, texture, wavelet-transformed, and LoG-derived features. All radiomic filtering and feature selection were fitted within the training partition only. Training-set reproducibility filtering based on repeated-segmentation data retained 876 stable descriptors with ICC > 0.85, corresponding to 66.6% of the initially extracted radiomic features. Redundancy pruning using a Pearson correlation threshold of |r| ≥ 0.90 reduced the feature set to 187 candidate descriptors. LASSO logistic regression with nested cross-validation then selected 42 radiomic predictors for downstream radiomics-only and early-fusion modeling.

**Figure 6:**
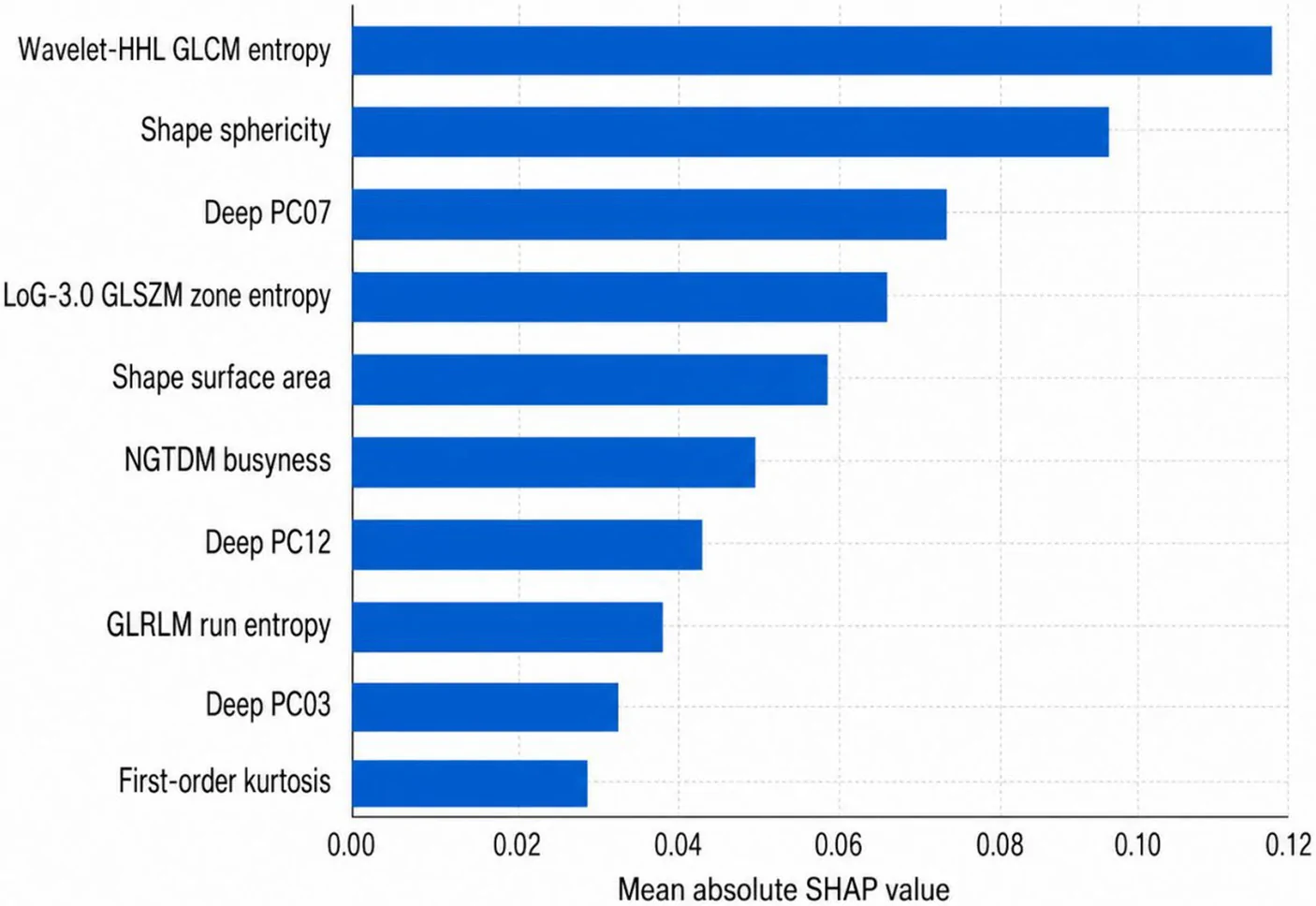
Top feature contributions in the integrated model.

The selected radiomic predictors were dominated by morphology- and heterogeneity-related features, including sphericity, surface area, entropy, run-length heterogeneity, zone entropy, and NGTDM busyness. These features are biologically plausible because aneurysm rupture status has been associated with surface irregularity, shape complexity, and heterogeneous wall- or sac-related imaging patterns (Kleinloog et al., 2018; Tanioka et al., 2020; Tomaszewski and Gillies, 2021). Feature contribution analysis of the integrated model showed that the leading contributors included both selected radiomic variables and PCA-compressed deep components, indicating that the fused model used complementary handcrafted and learned information rather than relying exclusively on one feature domain (Figure 6).

Feature contributions were summarized using mean absolute SHA*p* values from the integrated early-fusion XGBoost model. The highest-ranked contributors included both selected radiomic predictors and PCA-compressed deep-learning components, suggesting that the model used complementary handcrafted and learned imaging information. Radiomic features provide interpretable morphology and texture descriptors, whereas deep principal components represent compressed high-level spatial embeddings and should not be interpreted as direct anatomical biomarkers.

### 3.3 Segmentation reproducibility and uncertainty analysis

Repeated independent segmentation was performed in 80 patients, corresponding to 20% of the final modeling cohort. The repeated-segmentation subset preserved representation of rupture status and imaging modality where feasible. Inter-reader spatial agreement for aneurysm-sac delineation was high, with a mean Dice similarity coefficient of 0.89 ± 0.05 and a median Dice coefficient of 0.90 (IQR, 0.86–0.93). Feature-level reproducibility was also favorable, with a median ICC of 0.91 (IQR, 0.87–0.95) across the extracted radiomic descriptors. Overall, 876 of 1,316 radiomic features (66.6%) met the predefined reproducibility threshold of ICC > 0.85 and were retained for downstream redundancy pruning and LASSO selection. Among the 42 final LASSO-selected radiomic predictors, 39 features (92.9%) maintained ICC > 0.85, supporting the stability of most selected predictors under segmentation perturbation. However, a full prediction-level sensitivity analysis using alternate segmentation masks was not performed; therefore, the effect of segmentation variation on final predicted probabilities, AUC values, and selected feature composition remains incompletely characterized and is acknowledged as a limitation.

### 3.4 Deep learning feature analysis

The custom 3D residual CNN generated a 512-dimensional embedding for each CTA/MRA aneurysm-centered patch. The standalone CNN classification head was evaluated separately on the internal held-out test set and achieved an AUC of 0.861, indicating that the end-to-end deep-learning model captured rupture-status-related spatial information. However, its discrimination was lower than that of the PCA-compressed deep-embedding model evaluated with downstream gradient boosting, suggesting that frozen CNN-derived representations may provide more stable and reusable feature information than the standalone classification head alone in this cohort.

**Figure 7:**
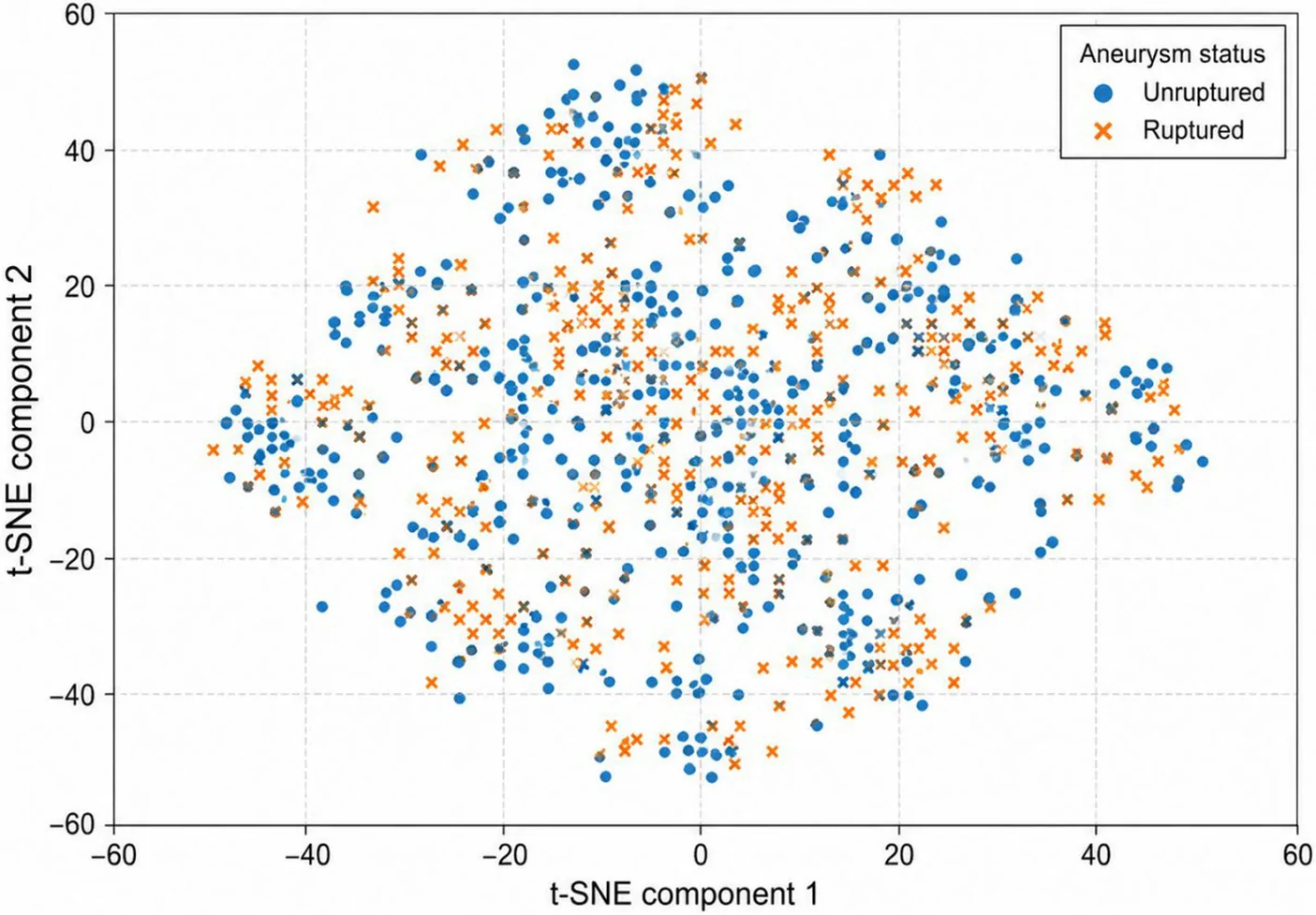
t-SNE visualization of deep-feature embeddings.

For qualitative visualization, t-SNE was applied to the 512-dimensional embeddings from the QC-passed final modeling cohort of 400 patients. The t-SNE projection was fitted without rupture-status labels and was not used for model training, feature selection, hyperparameter tuning, or performance evaluation. The resulting two-dimensional map showed partial local grouping but substantial overlap between ruptured and unruptured aneurysms (Figure 7). This pattern is consistent with the expectation that rupture-status discrimination relies on multivariable decision boundaries rather than simple visual separation in a two-dimensional embedding projection. Because t-SNE is sensitive to hyperparameter settings and preserves local neighborhood structure rather than global class boundaries, Figure 7 should be interpreted only as a qualitative representation of embedding structure. Quantitative model discrimination was assessed exclusively using empirical ROC analysis on the internal held-out test set.

The two-dimensional t-SNE projection was generated from 512-dimensional CNN embeddings extracted from the final modeling cohort of 400 patients. The projection was fitted without rupture-status labels and was used only for qualitative visualization. Partial local structure and substantial overlap were observed between ruptured and unruptured aneurysms. This visualization was not used for model training, feature selection, hyperparameter tuning, or performance evaluation.

### 3.5 Model performance

On the internal held-out test set, the integrated model combining 42 radiomic predictors and 128 PCA-compressed deep components achieved the highest overall performance among the evaluated models, with an AUC of 0.928, accuracy of 0.889, sensitivity of 0.902, specificity of 0.872, positive predictive value of 0.886, and negative predictive value of 0.889 (Table 4). The integrated model outperformed the radiomics-only model, which achieved an AUC of 0.884, and the deep-embedding model, which achieved an AUC of 0.903. The standalone residual CNN classifier achieved an AUC of 0.861, indicating that end-to-end deep classification captured rupture-status-related information but performed less favorably than the embedding-based models using downstream gradient boosting.

Pairwise AUC comparison using the DeLong test showed that the integrated model improved discrimination compared with the radiomics-only model (*p* < 0.01) and the deep-embedding model (*p* = 0.03). These findings suggest that early fusion provided complementary information beyond either handcrafted radiomic descriptors or learned deep embeddings alone. However, because the internal held-out test set included only 60 patients, including 24 ruptured and 36 unruptured cases, all performance estimates should be interpreted cautiously and require confirmation in an independent external cohort.

The ROC curves in Figure 5 were empirical curves generated from the binary rupture-status labels and model-predicted probability estimates in the internal held-out test set. The same held-out test-set labels and predicted probabilities were used to calculate the AUC values reported in Table 4. No schematic ROC curves, manually smoothed curves, or curves redrawn to match target AUC values were used. To support reproducibility and editorial/statistical verification, the de-identified internal held-out test-set labels and model-predicted probability estimates were retained as verification materials and can be provided to the editorial office or statistical reviewers upon reasonable request, subject to institutional data-sharing restrictions.

ROC curves were generated directly from the binary rupture-status labels and model-predicted probability estimates in the 60-case internal held-out test set. The same held-out test-set predictions were used to calculate the AUC values reported in Table 4. No schematic, manually smoothed, interpolated, or redrawn ROC curves were used.

The empirical ROC analysis showed that the integrated model achieved the highest discrimination on the internal held-out test set, with an AUC of 0.928, followed by the deep-embedding model with an AUC of 0.903 and the radiomics-only model with an AUC of 0.884. These curves were derived directly from the 60 internal held-out test cases, including 24 ruptured and 36 unruptured aneurysms. To support reproducibility and editorial/statistical verification, the de-identified internal held-out test-set labels and model-predicted probability estimates were retained as verification materials and can be provided to the editorial office or statistical reviewers upon reasonable request, subject to institutional data-sharing restrictions.

### 3.6 Calibration and decision analysis

**Figure 8:**
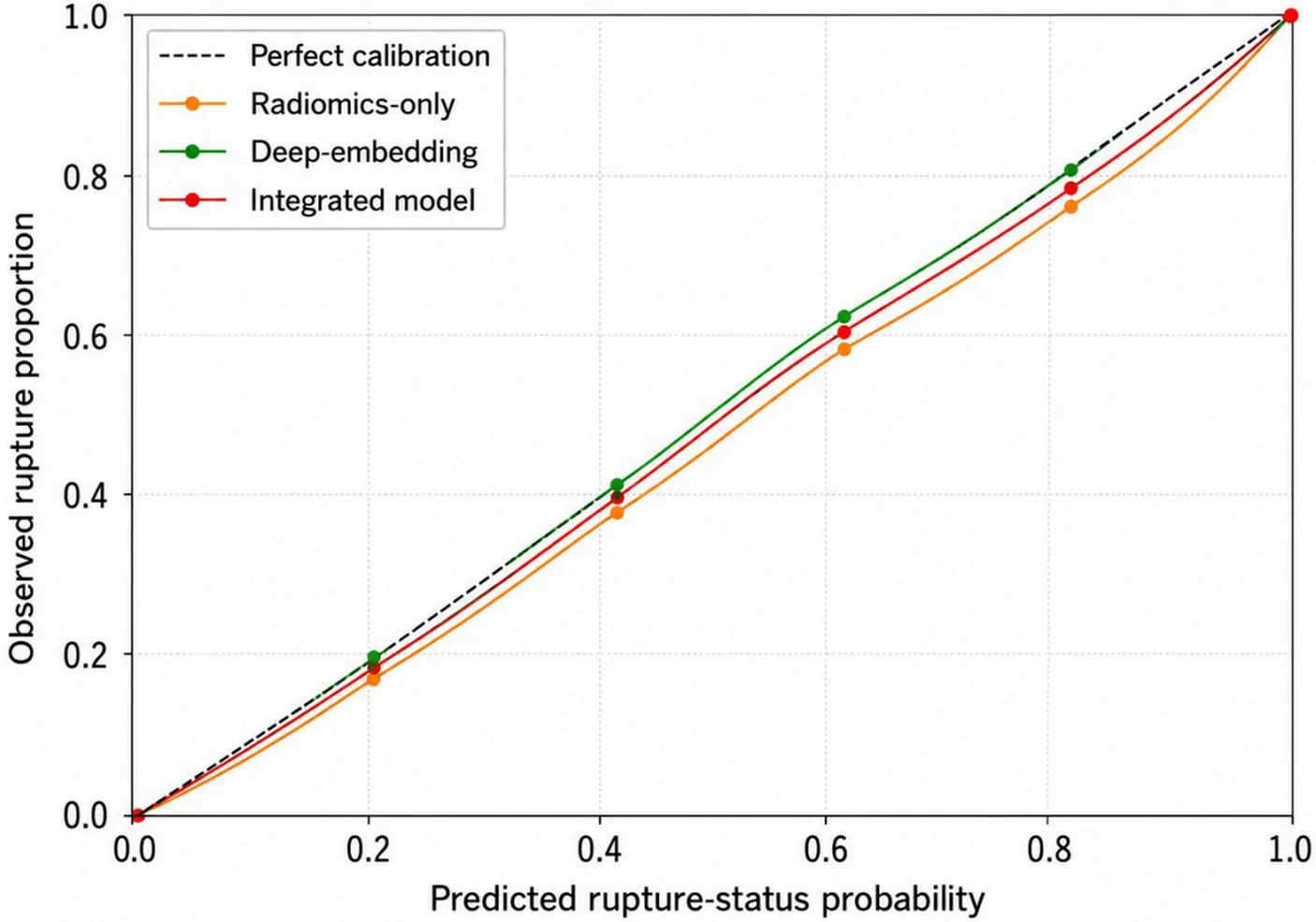
Calibration curves of the radiomics-only, deep-embedding, and integrated models on the internal held-out test set.

Calibration analysis showed that the integrated model produced well-aligned rupture-status probability estimates in the internal held-out test set (Figure 8). The calibration slope of the integrated model was 0.98, suggesting limited systematic overestimation or underestimation across the observed probability range. The integrated model also achieved the lowest Brier score among the evaluated models, with a value of 0.082 compared with 0.115 for the radiomics-only model and 0.101 for the deep-embedding model. These findings indicate that the early-fusion strategy improved not only discrimination but also probabilistic agreement between predicted rupture-status estimates and observed presentation labels in this internal test cohort.

**Figure 9:**
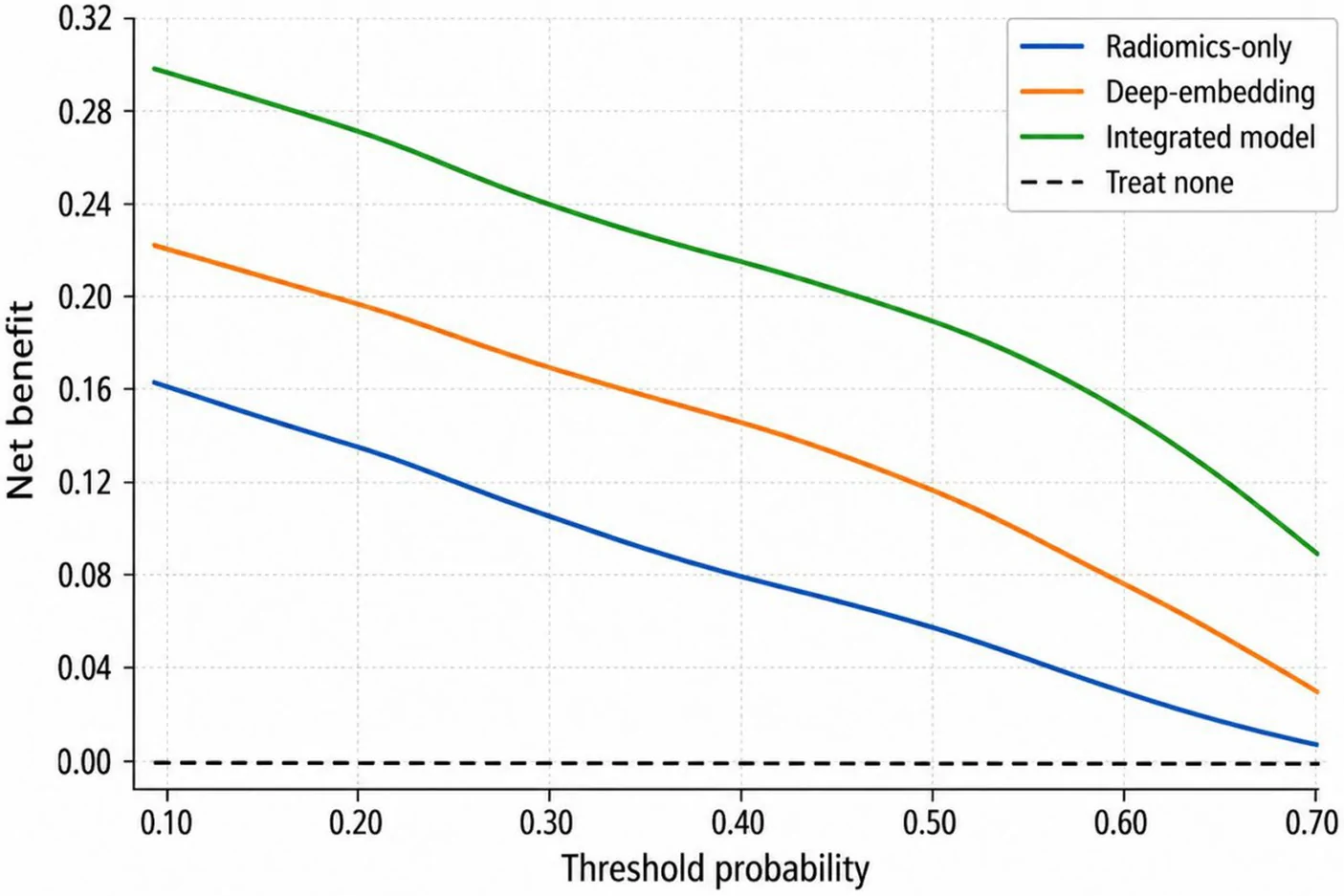
Decision-curve analysis of the radiomics-only, deep-embedding, and integrated models on the internal held-out test set.

Decision-curve analysis further showed that the integrated model provided higher net benefit than the radiomics-only and deep-embedding models across the prespecified exploratory threshold range of 0.10–0.70 (Figure 9). Within the threshold range of 0.30–0.60, the integrated model maintained consistently higher average net benefit than the single-domain comparators. These results suggest that combined radiomic and deep-learning representations may provide additional value for future imaging-based decision-support research. However, because the analysis was performed in an internal held-out test set and rupture status was defined at clinical presentation, these threshold probabilities should not be interpreted as clinically validated treatment, intervention, or surveillance thresholds. Prospective external validation and clinical-impact evaluation are required before any threshold-based clinical application can be proposed.

Calibration curves compare predicted rupture-status probability estimates with observed rupture-status proportions. The diagonal reference line represents perfect calibration. The integrated model showed the most favorable calibration profile, with a calibration slope of 0.98 and a Brier score of 0.082, compared with Brier scores of 0.115 for the radiomics-only model and 0.101 for the deep-embedding model.

Decision-curve analysis was performed across threshold probabilities from 0.10 to 0.70 in the internal held-out test set. The integrated model showed higher net benefit than the radiomics-only and deep-embedding models across most of the evaluated threshold range, particularly between 0.30 and 0.60. These thresholds were evaluated for internal exploratory purposes only and should not be interpreted as clinically validated decision thresholds.

## 4 Discussion

### 4.1 Principal findings

In this multicenter retrospective study, we developed and internally evaluated an imaging-based artificial intelligence framework for intracranial aneurysm rupture-status assessment using CTA/MRA-derived quantitative features. After cohort screening and preprocessing quality control, the final modeling cohort included 400 patients with 400 index aneurysms, comprising 160 ruptured and 240 unruptured aneurysms. The integrated early-fusion model combined 42 LASSO-selected radiomic predictors with 128 PCA-compressed deep-learning components extracted from a custom 3D residual CNN. On the internal held-out test set, the integrated model achieved the highest discrimination among all evaluated configurations, with an AUC of 0.928, outperforming the radiomics-only model, the deep-embedding model, and the standalone residual CNN classifier.

The results suggest that handcrafted radiomic descriptors and CNN-derived deep embeddings capture complementary aspects of aneurysm imaging phenotype. Radiomic features provide interpretable quantitative descriptors related to aneurysm shape, intensity distribution, and local texture heterogeneity, whereas deep embeddings may encode higher-order spatial representations that are not fully captured by predefined feature families. The superior performance of the integrated model supports the methodological value of combining engineered and learned representations in neurovascular imaging analysis. This finding is consistent with the broader aim of artificial intelligence and deep learning for neural data analysis, in which structured imaging features and data-driven representations can be integrated to characterize clinically relevant neurovascular phenotypes.

Importantly, the present study should be interpreted as an internal rupture-status assessment study rather than a prospective rupture-risk prediction study. The outcome label reflected rupture status at clinical presentation, and the model outputs were rupture-status probability estimates generated from CTA/MRA imaging data. Therefore, the model should not be interpreted as predicting future aneurysm rupture in untreated patients or as providing a clinically validated basis for treatment selection. The observed discrimination, calibration, and decision-curve findings provide methodological evidence that the proposed framework may support future research in quantitative aneurysm imaging, but independent external validation and prospective clinical evaluation are required before clinical implementation can be considered.

### 4.2 Comparison with previous studies

Previous imaging-based studies of intracranial aneurysms have commonly focused on morphological measurements, hemodynamic simulations, radiomic descriptors, or deep-learning classification models. These approaches have provided important evidence that aneurysm rupture status is associated with quantitative imaging phenotypes, including aneurysm size, shape irregularity, wall-adjacent signal patterns, and texture heterogeneity. However, single-domain approaches may capture only part of the complex imaging information related to rupture presentation. Handcrafted radiomic features are relatively interpretable but are constrained by predefined feature families, whereas deep-learning models can learn higher-order spatial patterns but may be less transparent and more sensitive to cohort size, imaging heterogeneity, and training instability.

The present study extends this line of research by integrating radiomics and deep-learning embeddings within a leakage-aware early-fusion framework. Several methodological features strengthen the internal validity of the analysis. First, all preprocessing, feature filtering, LASSO selection, PCA fitting, CNN training, and hyperparameter tuning were performed without using the internal held-out test set. Second, patient-level splitting was applied to avoid information leakage from patients with multiple aneurysms. Third, radiomic feature robustness was explicitly evaluated using repeated segmentation, and only reproducible features were allowed to enter downstream selection. Fourth, the final model was evaluated not only by discrimination but also by calibration and decision-curve analysis, providing a broader assessment of model behavior in the internal test cohort.

The superior performance of the integrated model suggests that aneurysm rupture-status assessment may benefit from combining interpretable radiomic descriptors with learned deep representations. The radiomics branch may reflect measurable geometry and intensity heterogeneity within the segmented aneurysm sac, while the CNN-derived embedding branch may encode spatial patterns that are difficult to prespecify manually. Importantly, the improvement of the integrated model over both the radiomics-only and deep-embedding models indicates that the gain was not simply attributable to using a more complex classifier, but to the complementary information provided by the two feature domains. This supports the methodological relevance of multimodal feature fusion for artificial intelligence and deep learning applications in neural and neurovascular data analysis.

Nevertheless, direct comparison with previous studies should be made cautiously. Differences in cohort composition, rupture-status definition, imaging modality, segmentation strategy, feature-selection pipeline, model architecture, and validation design can substantially influence reported performance. In addition, the present model was internally validated and should be regarded as a methodological framework for rupture-status assessment rather than a clinically deployable decision tool. External validation across independent institutions, scanner protocols, and patient populations remains necessary before the robustness and generalizability of the proposed approach can be established.

### 4.3 Clinical and research implications

The proposed framework has potential implications for future neurovascular imaging research and decision-support development. Intracranial aneurysm rupture assessment currently depends on clinical presentation, aneurysm morphology, imaging interpretation, and physician judgment. Quantitative imaging models may provide an additional layer of information by extracting high-dimensional patterns from routinely acquired CTA/MRA data. In this study, the integrated model generated rupture-status probability estimates that showed favorable discrimination, calibration, and exploratory decision-curve performance in the internal held-out test set. These findings suggest that combined radiomic and deep-learning representations may help characterize rupture-associated imaging phenotypes beyond conventional size and location descriptors.

From a research perspective, the study provides a reproducible computational workflow for aneurysm-centered image preprocessing, segmentation, radiomic feature extraction, CNN-based representation learning, early-fusion modeling, and internal validation. The model was developed using leakage-aware procedures, including patient-level data splitting, training-only feature selection and PCA fitting, and test-set evaluation only after all model-development steps had been completed. Such design principles are important for artificial intelligence and deep learning studies using neural and neurovascular imaging data, particularly when sample sizes are moderate and imaging protocols differ across centers.

Clinically, the present model should be viewed as a candidate research tool rather than a deployable diagnostic or treatment-selection system. The output represents a rupture-status probability estimate at clinical presentation, not a prospective prediction of future aneurysm rupture. Therefore, it should not be used to determine intervention, surveillance intervals, or patient counseling without further validation. Future studies should evaluate whether the proposed framework remains robust across external institutions, scanner vendors, imaging protocols, aneurysm locations, and patient populations. Prospective studies are also needed to determine whether model outputs provide incremental value over established clinical and morphological risk factors and whether they can improve multidisciplinary decision-making in a safe and interpretable manner.

### 4.4 Limitations

Several limitations should be acknowledged. First, although this was a multicenter retrospective study, model evaluation was limited to an internal held-out test set. No independent external validation cohort was available. Therefore, the reported discrimination, calibration, and decision-curve findings should be interpreted as internal validation results rather than evidence of generalizable clinical performance. External validation across independent institutions, scanner vendors, acquisition protocols, and patient populations is required before the proposed framework can be considered robust.

Second, the study assessed aneurysm rupture status at clinical presentation rather than prospective future rupture risk. The model output represents a rupture-status probability estimate derived from CTA/MRA imaging data and should not be interpreted as predicting future aneurysm rupture in untreated patients. This distinction is important because rupture-status classification and longitudinal rupture-risk prediction are related but methodologically different tasks. In addition, aneurysm morphology and local imaging appearance may be altered by the rupture event itself, introducing potential reverse-causality bias in retrospective ruptured-versus-unruptured comparisons.

Third, ruptured and unruptured aneurysms differed in known clinical and anatomical factors, including age, sex distribution, aneurysm size, and location. Although the imaging models were developed using patient-level splitting and leakage-aware training procedures, residual confounding cannot be excluded. The model may therefore partly reflect rupture-associated imaging phenotypes that are correlated with established clinical or anatomical risk factors rather than independent causal markers of rupture biology.

Fourth, segmentation uncertainty was evaluated at the spatial and feature-reproducibility levels but not at the full prediction level. Repeated segmentation in 80 patients showed favorable spatial agreement and feature-level reproducibility, and reproducibility filtering was applied before downstream feature selection. However, a full prediction-level sensitivity analysis using alternate segmentation masks was not performed. Therefore, the extent to which segmentation variability may affect final model probabilities, AUC values, calibration, decision-curve net benefit, or selected feature composition remains incompletely characterized.

Fifth, baseline descriptive analysis was restricted to the complete-covariate subset of 312 patients because 88 patients had missing non-imaging baseline variables, primarily hypertension and smoking history. These missing non-imaging variables were not used as inputs for radiomics, deep-learning, or integrated model development and did not affect the final modeling cohort of 400 patients. Nevertheless, the descriptive comparison in Table 5 should be interpreted as a complete-covariate subset analysis rather than a full-cohort baseline analysis.

**Table 5:** Baseline demographics and aneurysm characteristics in the complete-covariate subset.

Variable	Overall (*n* = 312)	Ruptured (*n* = 128)	Unruptured (*n* = 184)	*p* value
Age, years, mean ± SD	56.8 ± 12.3	58.9 ± 11.4	55.3 ± 12.8	0.018
Female sex, *n* (%)	170 (54.5)	79 (61.7)	91 (49.5)	0.041
Maximum aneurysm diameter, mm, mean ± SD	6.6 ± 3.4	8.6 ± 3.7	5.2 ± 2.4	<0.001
Location — MCA, *n* (%)	101 (32.4)	32 (25.0)	69 (37.5)	0.023
Location — ICA, *n* (%)	87 (27.9)	28 (21.9)	59 (32.1)	0.049
Location — ACom, *n* (%)	67 (21.5)	40 (31.3)	27 (14.7)	0.002
Location — PCom, *n* (%)	37 (11.9)	18 (14.1)	19 (10.3)	0.312
Location — posterior circulation, *n* (%)	20 (6.4)	10 (7.8)	10 (5.4)	0.394
Hypertension, *n* (%)	143 (45.8)	67 (52.3)	76 (41.3)	0.058
Smoking history, *n* (%)	94 (30.1)	45 (35.2)	49 (26.6)	0.088

Values are reported for the complete-covariate subset of the final modeling cohort. The final modeling cohort included 400 patients with 400 index aneurysms, whereas 312 patients had complete demographic, clinical, and aneurysm-level variables for uniform descriptive comparison. Eighty-eight patients had at least one missing non-imaging baseline covariate and were excluded from this descriptive table only. These missing covariates were not used as imaging-model inputs and did not affect radiomics extraction, deep-learning embedding generation, model training, validation, or internal held-out test evaluation. *P* values compare ruptured and unruptured groups within the complete-covariate subset. MCA, middle cerebral artery; ICA, internal carotid artery; ACom, anterior communicating artery; PCom, posterior communicating artery; SD, standard deviation.

Sixth, the internal held-out test set included only 60 patients, including 24 ruptured cases and 36 unruptured cases. Although bootstrap confidence intervals were reported, the modest test-set size limits the precision and stability of performance estimates. The deep-learning component may also remain vulnerable to overfitting because the number of training cases was limited relative to the complexity of volumetric neural representations.

Seventh, imaging heterogeneity may have influenced feature extraction and model behavior. Although standardized preprocessing, resampling, intensity normalization, and patient-level splitting were applied, CTA and MRA were acquired across multiple centers and scanners. Differences in scanner hardware, acquisition parameters, contrast timing, reconstruction kernels, and modality-specific signal characteristics may affect radiomic features and CNN-derived embeddings. DSA was used only as reference information for anatomical confirmation and rupture-status adjudication when available, but DSA images were not used as direct radiomic or deep-learning model inputs. Larger studies with harmonization strategies and modality-stratified external validation are needed (Table 6).

**Table 6:** Contextual comparison of model performance with selected previously published AI/radiomics studies on intracranial aneurysm rupture-status assessment.

Study	Modality/feature type	Sample size/validation context	Model or method	AUC	Accuracy	Sensitivity	Specificity
This study	Radiomics + deep-learning early fusion; CTA/MRA	400 patients; internal held-out test set	Integrated XGBoost model	0.928	0.889	0.902	0.872
Feng et al. (2023)	Deep learning + radiomics/morphology; CTA	898 aneurysms for training; 253 aneurysms for external testing	SVM, RF, and MLP classifiers	0.85–0.88	0.85–0.86	NR	NR
Xie et al. (2023)	CNN features + radiomics + clinical features; CTA	Original retrospective cohort	LASSO-selected features with SVM classifier	0.891	0.898	NR	NR
Yang et al. (2023)	Radiomics + machine learning	Original retrospective cohort	Multiple machine-learning algorithms; best-performing AdaBoost model	0.889	NR	0.716	NR
Ye et al. (2024)	Clinical-radiomics model; CTA	293 patients with small intracranial aneurysms	Clinical-radiomics nomogram	0.85	NR	NR	NR
Turhon et al. (2023)	Clinical, morphological, and radiomic multi-omics features	Original retrospective cohort with external validation	Deep-learning Transformer model	0.876	NR	NR	NR
Sohrabi-Ashlaghi et al. (2024)	Radiomics-based models	Systematic review and meta-analysis	Radiomics model synthesis	>0.80	NR	NR	NR
Zhang et al. (2026)	AI algorithms using CTA/DSA imaging data	Systematic review and meta-analysis	Imaging-based AI model synthesis	NR	NR	NR	NR
Study	Modality/feature type	Sample size/validation context	Model or method	AUC	Accuracy	Sensitivity	Specificity

All numerical values were extracted from published studies. NR indicates unreported or could not be consistently extracted from published abstracts. Direct numerical comparisons were not possible due to differences in cohort composition, aneurysm location, imaging modality, segmentation strategy, validation design, outcome definition, and reporting format. This study reports the performance of the internal held-out test, not the performance of independent external validation.

Finally, only saccular aneurysms were included, limiting generalizability to fusiform, dissecting, mycotic, previously treated, or recurrent aneurysms. These limitations mean that the present model should be regarded as an internally validated research framework for rupture-status assessment rather than a clinically deployable tool. Further external validation, prospective longitudinal assessment, and prediction-level segmentation-sensitivity analysis are required before any threshold-based recommendation for treatment or surveillance can be justified.

### 4.5 Future directions

Future studies should first validate the proposed framework in independent external cohorts. Such validation should include data from different institutions, scanner vendors, acquisition protocols, and patient populations to determine whether the integrated model remains robust under real-world imaging variability. Modality-stratified evaluation is also warranted because CTA and MRA differ in contrast mechanism, spatial resolution, intensity characteristics, and susceptibility to acquisition-related artifacts. Harmonization strategies, including scanner-aware preprocessing, feature harmonization, and domain-adaptation methods, may further improve model generalizability.

Prospective longitudinal studies are also needed to determine whether rupture-associated imaging features identified in this retrospective rupture-status assessment task have value for future rupture-risk stratification. The present model was trained to distinguish ruptured from unruptured aneurysms at clinical presentation; therefore, its outputs should not be interpreted as prospective rupture-risk estimates. Future work should include longitudinal follow-up of initially unruptured aneurysms, standardized clinical endpoints, and time-to-event modeling to evaluate whether radiomic and deep-learning features provide incremental prognostic information beyond established clinical and morphological risk factors.

Further technical development should focus on segmentation uncertainty and model interpretability. Although repeated segmentation demonstrated favorable spatial agreement and feature-level reproducibility, prediction-level sensitivity analysis using alternate segmentation masks was not performed in the present study. Future studies should quantify how segmentation variation affects predicted probabilities, calibration, feature selection, and decision-curve net benefit. In addition, interpretable modeling approaches should be developed to link influential radiomic features and deep-learning representations with anatomically and pathophysiologically meaningful aneurysm characteristics.

Finally, clinical translation will require rigorous evaluation beyond model discrimination. Future research should assess calibration stability, threshold behavior, clinical utility, workflow integration, reader-model interaction, and potential effects on multidisciplinary decision-making. Rather than replacing expert clinical judgment, imaging-based artificial intelligence tools should be evaluated as adjunctive systems that may help organize quantitative information and support transparent, evidence-informed discussion in neurovascular care.

## 5 Conclusion

This multicenter retrospective study developed an integrated artificial intelligence framework for CTA/MRA-based intracranial aneurysm rupture-status assessment by combining handcrafted radiomic features with CNN-derived deep-learning embeddings. In the final modeling cohort of 400 patients with 400 index aneurysms, the early-fusion XGBoost model achieved the best internal held-out test performance, with an AUC of 0.928, favorable calibration, and higher exploratory decision-curve net benefit than the radiomics-only and deep-embedding models. These findings suggest that radiomic descriptors and learned deep representations provide complementary information for characterizing rupture-associated aneurysm imaging phenotypes.

The proposed framework contributes a leakage-aware and reproducible methodological pipeline for neural and neurovascular imaging analysis, including standardized preprocessing, segmentation reproducibility assessment, radiomic feature selection, deep-feature extraction, early-fusion modeling, and internal validation. However, the study evaluated rupture status at clinical presentation and should not be interpreted as predicting future aneurysm rupture risk. Because no independent external validation cohort was available, the findings should be regarded as internally validated methodological evidence rather than proof of clinical deployability. External validation, prospective longitudinal evaluation, and prediction-level segmentation-sensitivity analysis are required before the model can be considered for clinical decision-support applications.

## Data Availability

The original contributions presented in the study are included in the article/Supplementary Supplementary material, further inquiries can be directed to the corresponding authors.
